# Neural predictors of hidden, persistent psychological states at work

**DOI:** 10.1073/pnas.2504382122

**Published:** 2025-10-13

**Authors:** Bear M. Goldstein, Shannon M. Burns, Ashley L. Binnquist, Macrina C. Dieffenbach, Csaba Konkoly, Shira Abramowitz, Matthew D. Lieberman

**Affiliations:** ^a^Department of Psychology, University of California, Los Angeles, CA 90095; ^b^Department of Psychological Science and Neuroscience, Pomona College, Claremont, CA 91711; ^c^Hum Capital, Los Angeles, CA 90272; ^d^Tiller Partners, Los Angeles, CA 90272; ^e^Summit Impact, Summit Series L.L.C., Cambridge, MA 02139

**Keywords:** psychological lenses, neural synchrony, fNIRS, machine learning, default mode network

## Abstract

Common workplace challenges such as feeling overwhelmed, burned out, or disengaged often remain hidden due to fear of judgment or social norms, contributing to rising mental health crises and organizational dysfunction. This study presents a brain-based framework for predicting these hidden and persistent psychological states through noninvasive neuroimaging. We used functional near-infrared spectroscopy to record neural activity from 67 executives in the field as they watched a video about workplace attitudes. We then applied a multitimepoint pattern analysis (MTPA) approach to reduce timeseries dimensionality and successfully classify whether individuals were feeling overwhelmed (72.8% accuracy) or in need of a new or different challenge (79.1% accuracy) in their careers using the temporal parietal junction (TPJ) and dorsal medial prefrontal cortex (dmPFC), respectively. The MTPA framework also allowed us to reverse-engineer specific thematic properties of the stimulus that evoked differential neural responses linked to these predicted outcomes. Emotional content in the video (e.g., reported distress) corresponded to the selected TPJ features that predicted whether someone was overwhelmed, while socially relevant content (e.g., missing social gatherings) aligned with the selected features from the dmPFC that predicted the need for a new or different challenge. These findings demonstrate the ability of neural measures to unobtrusively identify hidden and persistent psychological states in real-world settings, enabling targeted interventions that can improve well-being and engagement.

## Significance

Persistent psychological states shape how we interpret and navigate everyday experiences. At work—where most adults spend a significant portion of their lives—negative psychological “lenses” are not only widespread, but often remain hidden, limiting opportunities for support and intervention. This study presents a framework that uses noninvasive neuroimaging to predict the hidden and persistent subjective states through which individuals experience their careers. By uncovering these lenses in the field, our findings suggest that brain-based measures can offer insights into workplace experiences, highlighting their potential value for interventions aimed at fostering supportive and engaged environments. While demonstrated here in the context of work, this framework may also be applicable to other domains in which persistent internal states shape lived experience.

We spend about a third of our adult lives working. While work can be a powerful source of achievement, motivation, and engagement, the complex social dynamics in organizations can also cause problems. But no matter how overwhelmed, disengaged, or burned out we may feel, when someone asks, “How’s it going?”, we typically just reply: “Good.” Whether due to poor self-reflection or fear of judgment, the things we share at work do not always match how we think and feel. This social–emotional opacity prevents others from understanding how we are doing and providing support, contributing not only to the rising workplace mental and physical health crisis but also to deteriorating team culture via absenteeism, decreased productivity and creativity, and personnel turnover. What if there was a way to unobtrusively detect hidden and persistent social–emotional states (e.g., being overwhelmed at work) so that we can better identify issues and target support to those who need it most? In this study, we offer a framework to measure and predict these states through noninvasive neuroimaging.

Recent advancements in neuroscience have enabled researchers to leverage the brain to predict psychological and behavioral outcomes (1, 2). However, predicting the hidden and persistent ways in which people see things more generally—that is, their psychological lenses—has been a greater challenge. This is because these responses are shaped not just by sensory inputs but also by a mix of past experiences, expectations, and motivations that create enduring subjective interpretations of those inputs (3). To account for this complexity, rather than determining how people are seeing something directly from their brain activity, we instead determine whether people are seeing something similarly or differently from one another by measuring their intersubject neural synchrony (i.e., the coupling of different people’s neural activity over time). Research shows that neural synchrony in certain brain regions correlates with shared ways of experiencing and interpreting information (3, 4). Therefore, if we can identify group-level neural signatures for a certain characteristic, then we can determine whether a new individual has that same characteristic depending on how similar their brain responses are to a reference group.

While this framework builds on the large and growing literature linking neural synchrony to shared processes such as narrative interpretation (5), communication (6), and clinical factors like autism (7), it differs in a key way. Prior studies primarily use a forward-inference approach, testing whether people who share certain characteristics show more similar brain responses. In contrast, we take a reverse-inference stance (8), leveraging patterns of neural similarity to predict an individual’s psychological characteristics. This particular framework, coined the *neural reference groups approach* (9), has been used to predict both state-like phenomena, including experimentally induced narrative perspectives (10, 11), as well as trait-like characteristics, including firmly held sociopolitical beliefs (9) and attribution bias (12).

We expand this approach to test whether we can uncover more subtle and socially significant psychological lenses that are powerful in shaping our perspectives yet often remain hidden in daily life. Specifically, we focus on feeling overwhelmed, disengaged, and burned out at work. In contrast to more permanent traits, ideological stances, or one-off experiences, people’s negative lenses toward their careers are 1) pervasive as they contribute to the widespread workplace mental health and well-being crisis (13), 2) consequential as they lead to personal and organizational detriments (14–17), and 3) elusive as they have a tendency to remain hidden (18, 19). They are also malleable and responsive to intervention (20–22), making them a particularly valuable target for prediction and change. We focus specifically on business executives since they are uniquely positioned at the intersection of individual and organizational well-being. Negative lenses like feeling overwhelmed, disengaged, or burned out are particularly common in leadership roles (23), with implications that have an outsized impact on the organization and its members (24).

To test this, we leveraged the superior portability, motion tolerance, affordability, and comfort of functional near-infrared spectroscopy (fNIRS; 25, 26) to scan 68 business executives as they watched a video about work in a real-world setting—an endeavor that would have been impossible with alternative neuroimaging methods. Based on the assumption that people’s work-related lenses shape how they see and experience related content and that these lenses can be measured at the level of the brain, we aimed to define group-level neural signatures for these lenses and then classify new people based on their neural similarity to the reference groups.

Following previous neural synchrony-based classification research (9, 10, 27), we analyzed activity across the mPFC and TPJ—default mode network (DMN) hubs accessible with fNIRS. We distinguish between the anterior and dorsal subregions of the mPFC given research that suggests the anterior mPFC (amPFC) is more involved in affective and self-referential processing, while the dorsal mPFC (dmPFC) is more involved in mentalizing and social cognition (28, 29). The TPJ is of particular interest because it is likely responsible for integrating nonsensory inputs into differential subjective construals (3), a process roughly analogous to the application of a psychological lens. To reduce the dimensionality of the timeseries from these ROIs, we also apply multitimepoint pattern analysis (MTPA; 30) which uses feature selection to choose and analyze only the timepoints that are most informative for category classification.

Given the established link between neural synchrony in these areas and shared ways of experiencing and interpreting information, we hypothesize that models trained on brain activity in the dmPFC, amPFC, and TPJ will predict how people think and feel about their careers—specifically, whether they feel overwhelmed, burned out, or in need of a new or different challenge. In an exploratory analysis, we also use the features selected from the MTPA approach to reverse-engineer the specific timepoints in the video stimulus that evoke distinct neural profiles between groups, offering insights into the connection between stimulus content, neural activity, and psychological lenses. By providing an objective, brain-based measure of persistent and hidden psychological states, the proposed framework can support deeper insights and targeted interventions that foster engagement, productivity, and well-being.

## Results

### Predicting Work-Related Lenses.

**Fig. 1.**
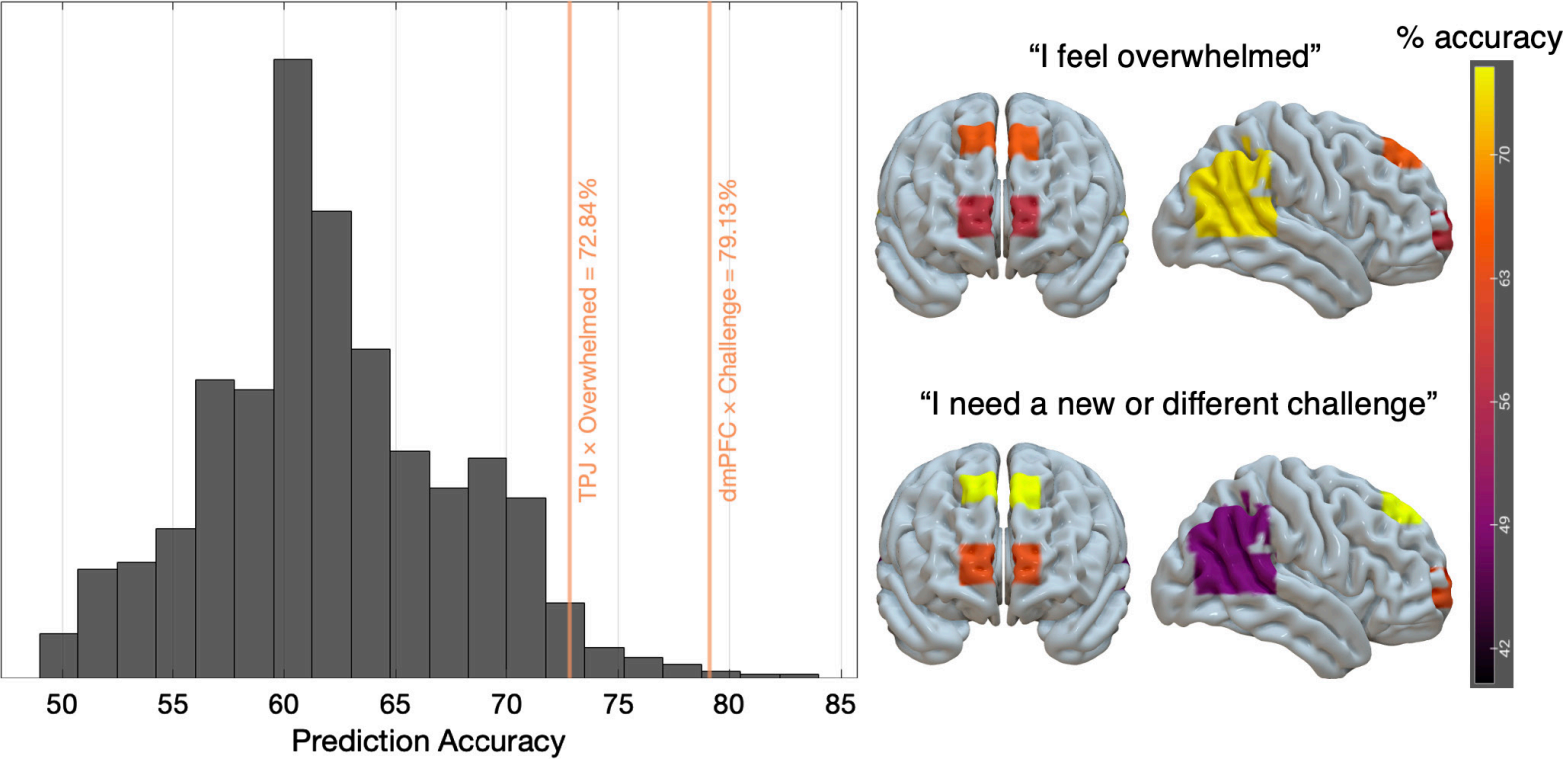
Results of maximal statistic permutation testing. The histogram shows the null distribution of prediction accuracies under permutation. For all possible ROI-outcome tests (nine total), we ran 1,000 permutations using the maximal statistic approach, shuffling labels and recording the highest accuracy across all ROI–outcome pairs per iteration to control for multiple comparisons. The vertical orange lines indicate observed prediction accuracies for the two most accurate tests—the TPJ predicting whether people feel overwhelmed and the dmPFC predicting whether people need a new or different challenge. These observed accuracies were statistically significant after maximal statistic correction (*P* = 0.035 and *P* = 0.002, respectively). The images on the *Right* show a heatmap of observed prediction accuracies overlaid on the brain for the “I feel overwhelmed” measure (*Top* two images) and the “I need a new or different challenge” measure (*Bottom* two images). Yellow areas reflect greater prediction accuracy.

We used an MTPA–neural reference groups approach to predict how people feel about their careers. Neural timeseries from the TPJ predicted whether people feel overwhelmed with 72.84% accuracy. Neural timeseries from the dmPFC predicted whether people feel like they need a new or different challenge with 79.13% accuracy. Both prediction accuracies were statistically significant under a maximal statistic permutation test that corrected for multiple comparisons across all ROI–outcome pairs (*P* = 0.035 and *P* = 0.002, respectively; see Fig. 1). The mean maximal statistic value was 62.13%; chance levels were approximately 50%. Together, these results suggest that we can use people’s neural activity to predict relevant social–emotional characteristics, such as how people are feeling about their careers, without having to explicitly ask them.

No other brain-outcome combinations produced statistically significant results after multiple comparison correction. The dmPFC predicted whether people feel overwhelmed at 64.51% accuracy, and the amPFC predicted whether people need a new or different challenge at 63.29% accuracy, but neither survived the maximal statistic permutation correction (*P* = 0.318 and 0.383, respectively). Classification accuracy levels for the “I feel burned out” measure were not significantly above-chance for any of the ROIs. Table 1 shows the prediction accuracies of the models for each measure and ROI.

**Table 1.** Average prediction scores and elastic net feature retention for each ROI and outcome measure

		Observed Accuracy %	Observed Features #
I feel overwhelmed	TPJ	72.84*	36.72
	dmPFC	64.51	28.95
	amPFC	57.94	20.46
I need a new/different challenge	TPJ	48.61	1.07
	dmPFC	79.13*	34.84
	amPFC	63.29	20.75
I feel burned out	TPJ	51.42	15.27
	dmPFC	49.11	12.27
	amPFC	55.94	10.06

Note: *P < 0.05 under maximal statistic permutation correction.

### MTPA Performance.

**Fig. 2.**
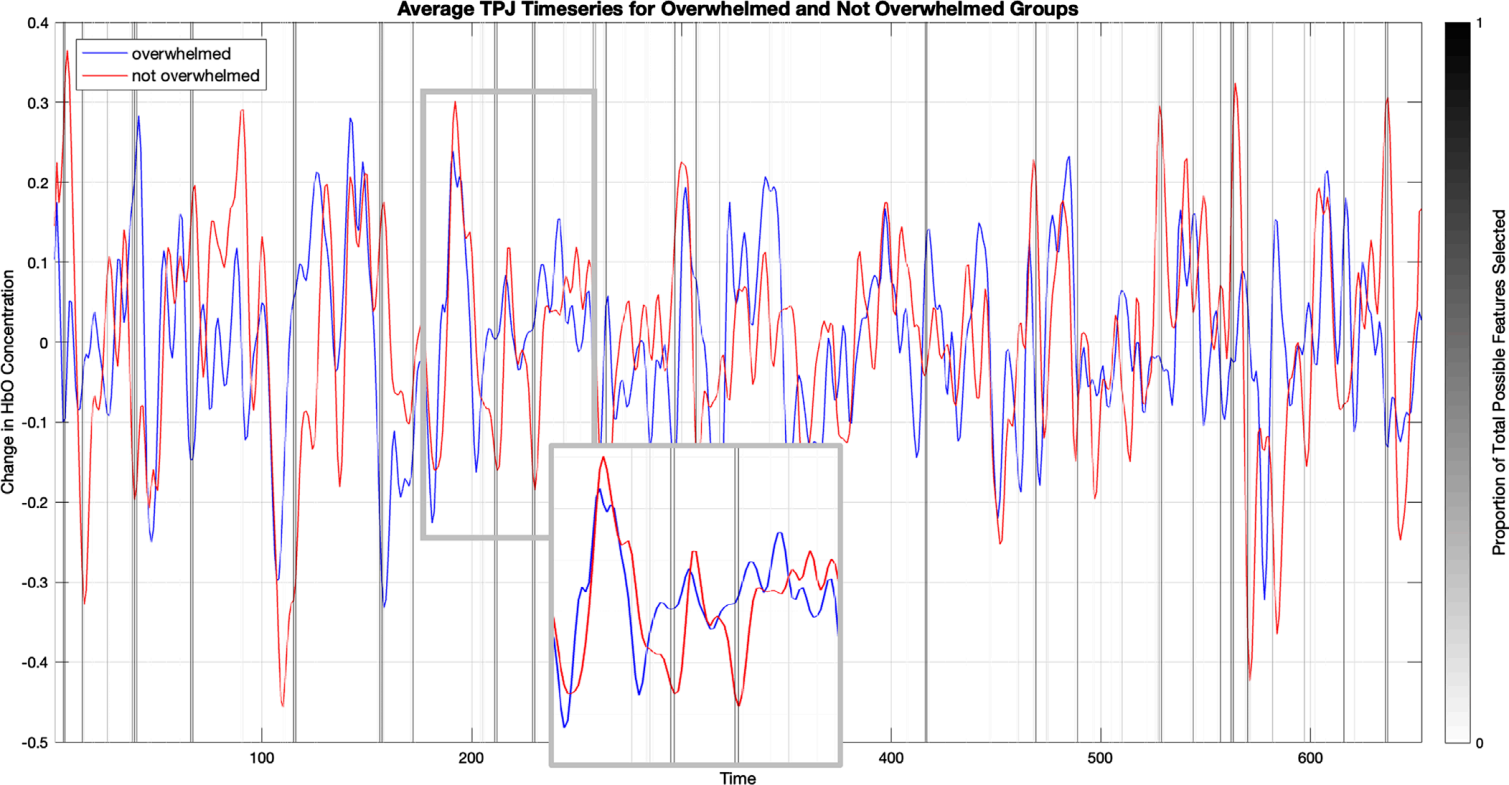
Example case of the elastic net feature selection. In this example, we are predicting whether people feel overwhelmed with the TPJ timeseries. The red and blue lines represent the average timeseries for the overwhelmed and not overwhelmed groups in the training set, and the vertical gray bars represent the features that the elastic net retained. The darkness of the vertical line represents the proportion of tests and iterations in which that feature was selected—a black line is a feature that was selected every time, and a middle-gray line was selected half the time. Upon inspection, it is apparent that the selected timepoints generally correspond to the points at which there are the biggest differences between the two groups’ timeseries.

We also examined the performance and impact of the MTPA approach using elastic net feature selection. After downsampling each timeseries to one sample per second, we were left with 653 timepoints. Considering our sample sizes of N = 67 and N = 62 depending on the outcome measure, the high number of features created an approximately 1:10 ratio of samples to features—the inverse of the 10:1 sample to feature ratio that is considered optimal for machine learning purposes (31, 32, cf. 33). The elastic net-based MTPA approach drastically reduced the number of features in the timeseries, which were subsequently used to train the logistic regression. Fig. 2 illustrates the function of the elastic net for the case of the TPJ predicting whether people are overwhelmed or not.

The MTPA retained the largest number of features for the two ROIs and questions that yielded accurate predictions (Table 1). The elastic net selected an average of 36.72 and 34.84 features across all tests and iterations when predicting whether participants needed a new or different challenge with the dmPFC and whether participants felt overwhelmed or not with the TPJ, respectively. Reducing from 653 timepoints to about 35 allowed for more efficient training and decreased risk of overfitting. In contrast, too few features were retained for the other area-outcome combinations that were not predictive, suggesting that these models struggled to converge on a reliable set of features.

To better understand the impact of MTPA, we ran an exploratory analysis using a ridge-penalized logistic model without feature selection. This classifier was trained and tested using the same cross-validation procedure as the main model, allowing us to isolate the impact of MTPA on predictive performance while ensuring a regularized non-MTPA baseline. When predicting whether someone feels overwhelmed using the full TPJ timeseries, the regularized classifier yielded a prediction accuracy of 51.2%. When predicting whether someone needs a new or different challenge with the dmPFC timeseries, the logistic regression classifier produced a prediction accuracy of 59.5%. MTPA improved our accuracy in these two analyses by 21.6% and 19.6%, respectively, which in our sample sizes of 67 and 62 participants translates to about 14 and 12 more people classified correctly for each measure.

### Reverse-Engineering Stimulus Efficacy.

Since MTPA selects for certain features—essentially timepoints within a timeseries that are locked to an extended, naturalistic video stimulus—we can examine what was occurring in the video when group-level neural responses diverged. To do this, we compared the probability that people were discussing a given topic or theme in the video during the highly selected timepoints versus timepoints that were not selected at all.

**Fig. 3.**
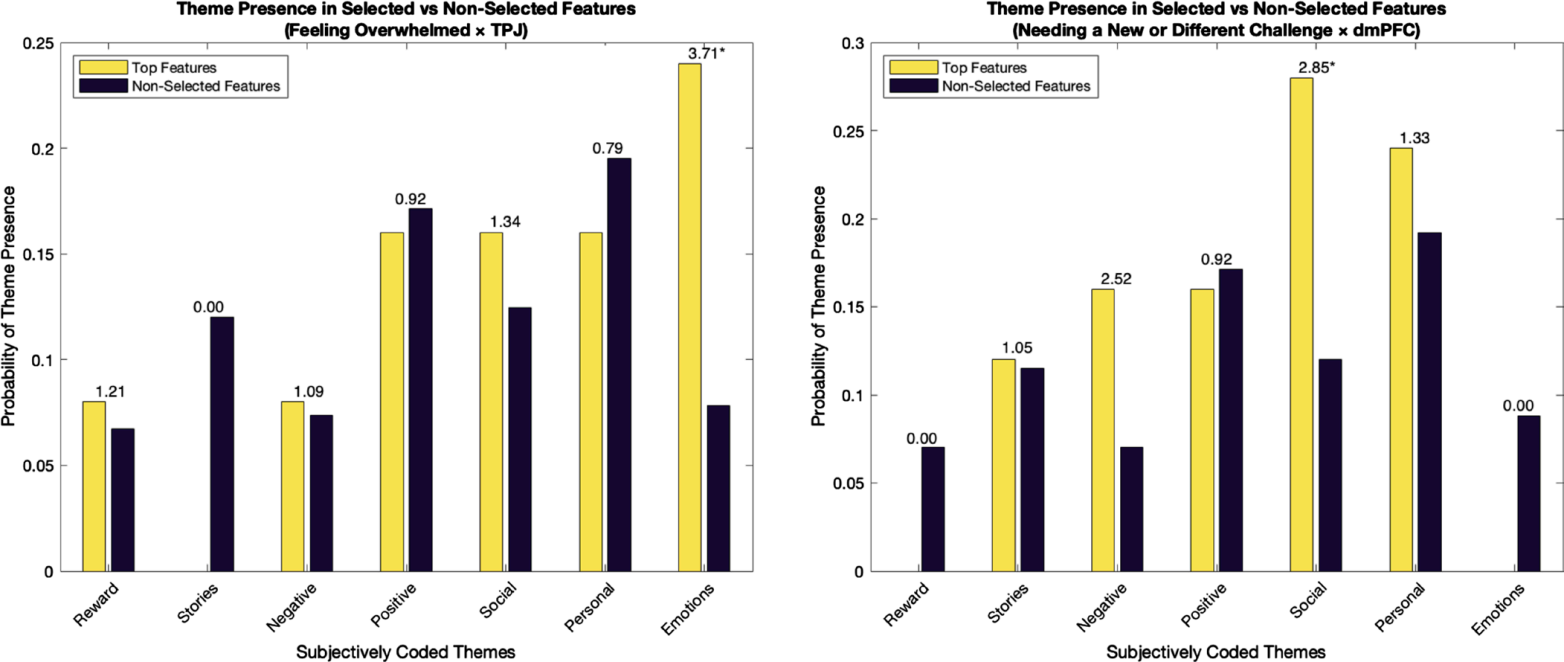
Probability of frequently selected features and nonselected features corresponding to themes from the video stimulus. The barplots show how likely it is for certain elements of the video stimulus to correspond to selected features versus features that were not selected by the MTPA. The *Left* plot illustrates the relationship between thematic elements and features selected when using the TPJ to predict whether people felt overwhelmed. Highly selected features were significantly associated with moments where speakers in the video were discussing emotions (odds ratio = 3.71, *P* = 0.017). The *Right* plot corresponds to the dmPFC as it predicts whether people need a new or different challenge. Each bar is associated with one of our subjectively coded video themes. Highly selected features were significantly associated with moments where speakers in the video were discussing social topics (odds ratio = 2.85, *P* = 0.024).

Odds ratios with circle-shift permutation tests revealed significant associations for two themes—emotional and social (Fig. 3). Selected timepoints from the TPJ as it predicted feeling overwhelmed were significantly associated with moments where the speakers in the video discussed their emotions (odds ratio = 3.71, *P* = 0.017), suggesting that when participants viewed emotion-related content their TPJ activity reliably differed depending on whether they felt overwhelmed or not. Additionally, selected timepoints in the dmPFC as it predicted needing a new or different challenge were significantly associated with moments where the speaker discussed social topics (odds ratio = 2.85, *P* = 0.024). This suggests that when participants watched parts of the video where people talked about the social aspects of work and their relationships (e.g., with clients, coworkers, or friends and family), their dmPFC activity differed depending on whether they needed a new or different challenge in their career. Together, these results indicate that emotional and social themes in the video were particularly salient in driving differential neural responses predictive of career-related lenses.

## Discussion

The purpose of this research was to use people’s neural activity to predict hidden and persistent psychological lenses in a real-world setting. We focused on negative feelings toward one’s career, such as feeling overwhelmed, burned out, or needing a new or different challenge. Grounded in the idea that brain activity can offer unique insights into people’s psychological lenses, we used tools from social neuroscience to provide a window into understanding, measuring, and predicting how people felt about their careers in an unobtrusive manner. Given that similar brain responses in areas responsible for sociocognitive functions like mentalizing, semantic processing, and self-referential thinking have been linked to similarities in how people “see” things (3, 34, 35), we hypothesized that models trained on neural activity from the TPJ, dmPFC, and amPFC could effectively predict people’s career-related sentiments.

To test this hypothesis, we went into the field to measure the brain activity of real-world executives as they watched a video about people’s work-related attitudes. In line with our hypotheses, these models were able to predict key aspects of how participants felt about their careers. Neural activity from the TPJ predicted whether participants felt overwhelmed with 72.84% accuracy, and dmPFC activity predicted whether participants felt like they needed a new or different challenge with 79.13% accuracy. Both results are significantly above chance, demonstrating the potential for neural markers to reveal complex and oft-hidden social–emotional experiences.

The predictive framework used here does not attempt to explain every possible source of neural difference (e.g., personality, background, neurodiversity). Instead, it leverages neural patterns to classify individuals into predefined groups. Future research can further dissect within-group heterogeneity by stratifying participants based on demographic or personality variables. Additionally, our sample size—while relatively large for a neuroimaging study conducted in the field—remains modest by machine learning standards. We addressed the poor initial sample-to-feature ratio challenges through dimensionality reduction via elastic net regularization, strict cross-validation, and maximal statistic permutation testing, but future studies with larger samples are needed to verify the stability and generalizability of these findings.

Notably, the MTPA approach allowed us to reduce the high temporal dimensionality of our neural timeseries prior to model training, which boosted prediction accuracy by 21.6% and 19.6% compared to ridge-penalized logistic regression with no feature selection. This dimension reduction was essential for preserving the richness of our extended naturalistic stimuli while decreasing the risk of overfitting.

MTPA also enhanced the interpretability of our models. By isolating key features and examining their respective timepoints in the video stimulus, the MTPA approach allowed us to identify which aspects of the video produced distinct neural responses across groups. For example, moments where speakers discussed their emotions aligned with timepoints where the TPJ was predictive of whether participants felt overwhelmed. This may reflect the emotional complexity of feeling overwhelmed—encompassing stress, frustration, pain, or sadness—which could evoke different neural resonance based on participants’ experiences. Emotionally coded content in the video during selected timepoints includes comments like, “[Firing people] is painful. It hurts. And, you know, we have to make it through it, and do those things, and be human about it. But that does not make it hurt any less.” On the other hand, social topics in the video were linked to timepoints from the dmPFC that predicted whether participants needed a new or different challenge. The social nature of one’s position at work (e.g., interacting with clients or work’s effect on one’s social life) may explain why people who need a new challenge respond differently to this content. Socially coded content during selected timepoints includes comments like, “I am not really social at all anymore. I have missed weddings. I have missed important events with my family.” These findings offer insight into how specific aspects of a stimulus can differentially affect neural activity depending on one’s psychological lenses. More broadly, this approach provides a potential strategy for identifying effective content and designing targeted future stimuli that drive neural differences across groups.

The patterns observed in our predictive models, along with the stimulus-specific features identified through MTPA, also provide tentative insights into how subregions of the DMN may support distinct psychological functions. According to the CEEing model (3), the TPJ supports effortless subjective construal by integrating sensory and contextual information, whereas mPFC areas are involved in more effortful, reflective forms of social reasoning. Our exploratory reverse-engineering analysis broadly aligns with this view—TPJ activity was predictive during broader emotional moments in the video, while dmPFC activity was more engaged during explicitly social reflections, such as interpersonal strain from poor work–life imbalance. This pattern may also reflect differences in the measures themselves: One can imagine that feeling overwhelmed may stem from more passive construal of experience, whereas recognizing a need for a new or different challenge may require more intentional and effortful reflection. Future research should test these interpretations to further clarify how DMN subregions contribute to distinct psychological lenses.

Other regions involved in affective processing, such as the dorsal anterior cingulate cortex, likely contribute to these experiences as well, but they fall outside the spatial coverage of fNIRS. Replication with functional magnetic resonance imaging (fMRI) would allow for a more comprehensive whole-brain assessment. The study’s experimental design—passively watching a video stimulus—is well suited to fMRI, though the field-based nature of the present study made such implementation impossible.

### Benefits for the Workplace and beyond.

This study builds on the theoretical framework of neural reference groups approaches (9–12) but extends it to focus on social–emotional experiences that are fluid, context-dependent, and directly relevant to real-world challenges. By using portable fNIRS in a field setting, we demonstrate that it is possible to predict common, consequential, and concealable social–emotional experiences in the field. Traditional implicit measures are limited by their detachment from real experience and are often influenced by demand characteristics (36), whereas neural synchrony can capture rich, unobstructed information predictive of people’s subjective states. While further validation in diverse settings and samples is necessary before broader application, the ability to predict implicit and nuanced social–emotional states could create additional avenues for helping organizations and teams foster happier, healthier, and more engaged cultures.

Although our findings represent only an initial proof of concept, one potential future application of this framework lies in understanding leadership experiences in high-stakes environments. Persistent stress from leadership roles can impair leaders’ well-being and ripple through the organization, affecting team morale, productivity, and decision-making. Given that we sampled directly from this population, this research provides preliminary evidence for a tool that can help leaders monitor whether they are overwhelmed or ready to move onto new challenges, providing insights to help them manage stress even when their own perspectives may be clouded by their dedication to a mission or team performance. Though speculative and requiring extensive safeguards, this kind of approach could also be used to assess team-level dynamics or evaluate the effects of different organizational interventions or initiatives intended to shift workplace dynamics. Other possible future directions include applying this framework to contexts like therapy or education, where understanding individuals’ internal states could help therapists identify unintentionally hidden outlooks and emotional “blindspots” of their patients, while educators could use this approach to understand how different students respond whether they grasp the material or not. Still, any such extensions would require substantial validation and should be considered exploratory at this stage.

While widespread application is not yet possible, it remains important to consider the potential for misuse as neural prediction tools evolve. One key area of concern, particularly in hierarchical environments like the workplace, is the potential for neural data to be used for discrimination. As these technologies grow, so must the ethical guardrails that guide their use, including robust transparency, consent, and privacy protections. The field of genetics offers some precedent for this dilemma: While genetic data were seen as a tool for understanding and improving health, it also posed risks for discriminatory practices, leading to the Genetic Information Nondiscrimination Act of 2008. A similar legal framework may be necessary to ensure neural data are used responsibly and solely to promote individual and organizational well-being.

### States, Traits, and Lenses.

The MTPA-neural reference groups approach offers a framework for studying a conceptually distinct level of mental states, bridging a gap between prior research on transient states and enduring traits. Numerous neuroimaging studies have engaged in “mindreading” by using multivariate decoding methods to identify what a person is seeing at a particular moment (37, 38). This can be used to identify whether a person is, for instance, looking at a face or a house, and it focuses on what is being experienced in this very moment based on concrete sensory stimuli. At the other of the spectrum, numerous studies have used brain data to identify the stable personality traits of individuals (39).

In contrast, our approach focuses on the overlooked middle ground between states and traits: psychological lenses. Psychological lenses reflect the subjective filters through which each of us effortlessly experiences the world in different ways. These lenses can be longstanding and influenced by our biological and psychological traits, but they are also influenced by the people around us and the situations we find ourselves in for extended periods of time. Someone might feel overwhelmed by a particular job or by being part of a toxic team and that could influence how a person sees everything related to work, but that lens can shift dramatically with a change in one’s responsibilities or a new assignment. When the MTPA-neural reference groups approach is applied to identify a lens, it is not decoding the objective content that a person is presented with, but rather the subjective experience of that content that differs from person to person, often reflecting an enduring way of seeing and experiencing the world.

### Conclusion.

We all go through difficult times. But for one reason or another, we do not always ask for the help we need or even know that we need to ask. The present research shows how neural activity can be used to detect hidden and persistent psychological lenses related to the context in which we spend a significant portion of our adult lives—work. By using the brain as a predictor, we can understand and predict real-world problems in a direct and meaningful way, offering insights for how to effectively communicate, connect, and care for one another.

## Materials and Methods

### Participants.

Participants (N = 68) were business executives who attended “Summit LA,” which is an organizational festival that invites successful founders, CEOs, and other executives to build community and discuss ideas for a weekend in Los Angeles. Of the 68 participants, 47 identified as male and 21 identified as female. The mean age was 41.5 y (SD = 9.87). One participant did not fill out the poststudy questionnaire and was excluded, resulting in a final sample of N = 67.

Participants were recruited on a volunteer basis, received a description of the study, provided informed consent, and were free to withdraw at any time. Data were collected by Resonance Inc. in collaboration with Summit LA, and only fully de-identified fNIRS and questionnaire records were transferred to the UCLA Social Cognitive Neuroscience Laboratory for analysis. The UCLA Institutional Review Board reviewed the project and determined that IRB oversight and approval for analysis on secondary, deidentified data were not necessary given a non-human-subjects research classification.

### Procedure.

**Fig. 4.**
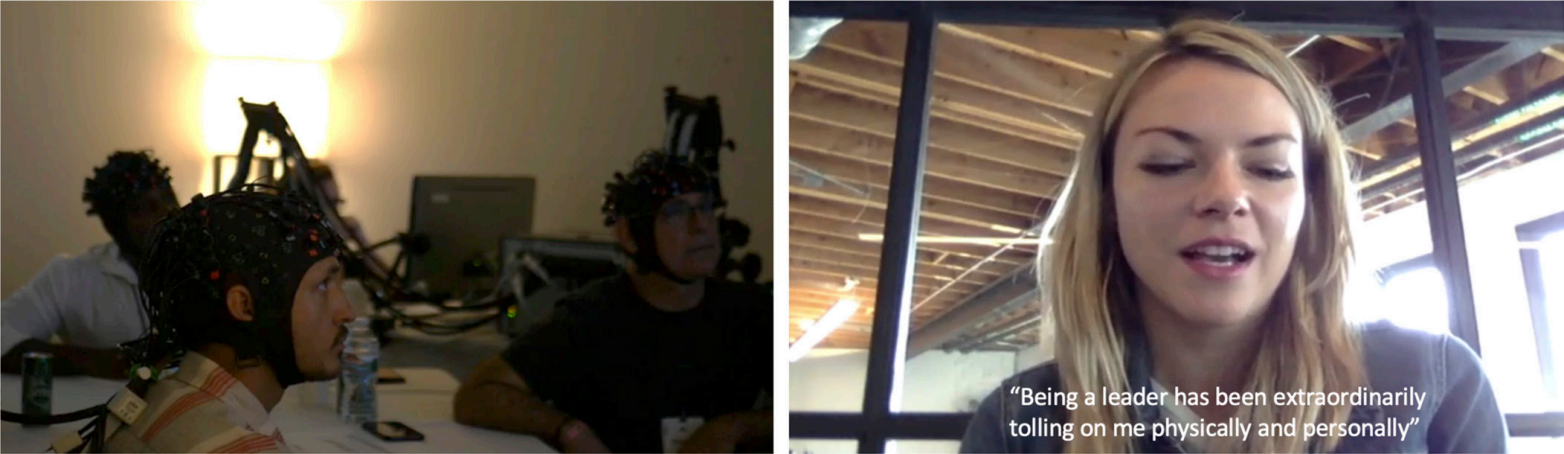
Stills from the experiment and video stimulus. The *Left* image shows participants wearing fNIRS and watching the video together. The *Right* image is a still from the video stimulus, where other business executives were instructed to speak about the positive and negative aspects of their work. Captions were not included in the original stimulus.

Consenting participants were invited into our pop-up neuroimaging lab in an office suite near the Summit LA festival and fitted with fNIRS. The experimental session consisted of two parts. First, participants engaged in a group interaction task where they took turns pitching each other ideas. The current study uses data from the second half of the session, unrelated to the group interaction task. In this phase of the experiment, participants’ brains were scanned with fNIRS while they watched a video compilation of other executives talking about their attitudes toward work (Fig. 4). The video featured executives talking about the positive and negative aspects of their work and their attitudes toward their careers. After the video, participants completed a questionnaire (*SI Appendix*) probing their thoughts and feelings toward their own career.

### Stimuli and Materials.

#### Video.

The video used to collect the neural timeseries data was a compilation of business executives candidly discussing their work and career-related sentiments. To create this video, we invited executives that were not attending Summit LA to record themselves discussing the positive and negative aspects of their work and their work–life relationship. The speakers recorded their responses on their personal computers, and we stitched them together into a video that totaled 10 min and 53 s.

#### Outcome measures.

After watching the videos, participants responded to four outcome measures that probed negative social–emotional sentiments toward their career: “I need a new or different challenge,” “I feel overwhelmed,” “I feel underappreciated,” and “I feel burned out.” Participants were instructed to respond to the measures as if they were thinking about their career in general. We used a seven-point Likert scale from “strongly disagree” to “strongly agree.”

We selected these measures because we believed them to be generally representative of aversive work-related experiences, but distinct enough from one another so that we could capture different elements of the phenomenon. Each measure reflects a different facet of aversive work-related lenses: Feeling overwhelmed captures the immediate, situational demands or stressors directly tied to ongoing experiences; feeling burned out reflects a broader, cumulative effect of prolonged exposure to stress and dissatisfaction; needing a new or different challenge probes a forward-looking, organizational dimension tied to engagement and growth; feeling underappreciated emphasizes the social and relational aspects of work. Interitem correlations were modest (r = 0.14 to 0.42, mean r = 0.32), and Cronbach’s α for the four outcome items was 0.66, below the conventional 0.70 criterion for combining items into composite measures (40). These results indicate that the items measure related yet distinct experiences. Accordingly, we analyzed each outcome separately rather than forming a composite (full details in *SI Appendix*).

### fNIRS Data Acquisition.

**Fig. 5.**
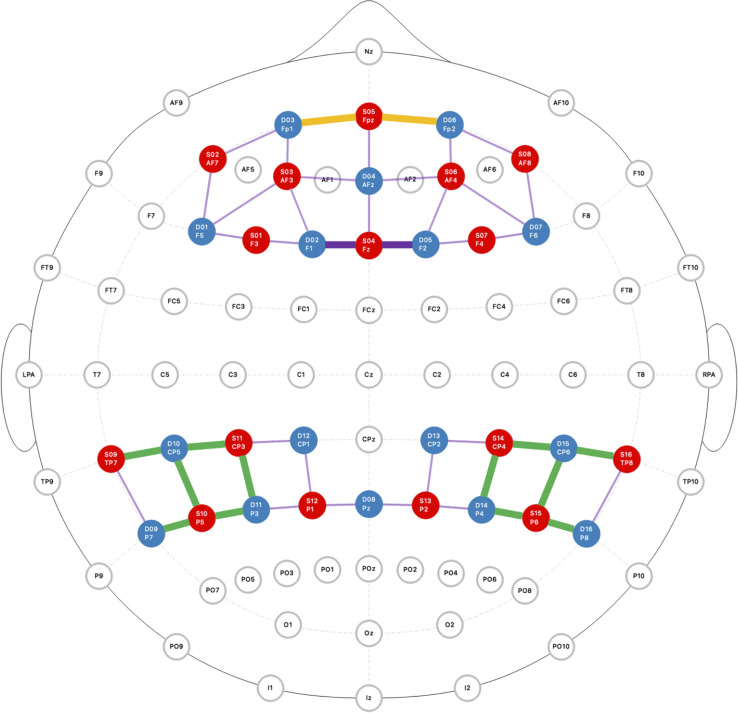
Montage of fNIRS probes across the prefrontal cortex, temporal parietal junction, and superior parietal lobule. The red dots represent source optodes, and the blue dots represent detector optodes. ROIs are highlighted in orange (amPFC), purple (dmPFC), and green (TPJ).

We used NIRx NIRScout machines (NIRx Medical Technologies, LLC, New York, USA) with a sampling rate of 3.906 Hz at wavelengths of 760 and 850 nm. Optodes on the fNIRS caps were arranged at 3-cm average source-detector separation distance over the PFC, the TPJ, and superior parietal lobule (Fig. 5). We chose these areas to measure activity in brain areas responsible for social cognition, attention, and subjective construal processes broadly (3, 29). Our montage also covered nonmentalizing regions (i.e., lateral PFC and the superior parietal lobule), which were not included in analyses as they did not fit our hypotheses.

A 10-10 UI external positioning system was used to standardize the placement of our probes across participants. Caps were placed on participants heads using anatomical landmarks including the nasion (the indentation between the forehead and the nose), inion (the bump at the lower rear of the skull), left and right preauricular points (just in front of the ears), and the vertex of the skull. Each participant wore a cap matched to their head circumference to ensure consistent fit. These procedures were deemed sufficient for maintaining alignment of optodes across individuals given the demands of our field-based setting. Channels could then be projected into MNI space using probabilistic modeling (41) to localize the ROIs within a common brain space.

### Data Preprocessing and Preparation.

#### Neural data.

fNIRS data were sent through a preprocessing pipeline (42) that utilized custom scripts along with scripts from the Homer2 software (43). For noisy and oversaturated channels, a modified quartile coefficient of dispersion (44) was used to remove any channel that exceeded the coefficient cutoff (Cthresh = 0.6 – 0.03*sampling rate) for 2 s or more. Further refinement of the data included corrections for motion and nonneural changes in blood oxygenation. To address motion artifacts within remaining channels, spikes greater than 5 SD change in less than 1 s were removed. To address nonneural physiological influences (e.g., cardiac and respiratory rhythms) and baseline drift, a conservative bandpass filter (0.008 to 0.2 Hz) was applied. Filtered data were then transformed from optical density to hemoglobin (HbO) concentration values using the modified Beer–Lambert Law with a standard differential path length filter. The final quality control step involved an autocorrelation change assessment to gauge the impact of motion correction. Channels displaying a substantial change in autocorrelation (exceeding a threshold of r = 0.1) were deemed significantly influenced by motion and excluded from subsequent analyses. The data were normalized by z-scoring. We specifically focused on oxygenated HbO, which offers a better signal-to-noise ratio (45), comparatively higher amplitude (46), and stronger correlations with fMRI BOLD signals than deoxygenated HbO (47), making it the most sensitive and statistically powerful index for our machine-learning purposes.

After preprocessing, several steps were then employed to reduce dimensionality and smooth the data during preprocessing. First, we selected specific neural ROIs: the bilateral TPJ, the amPFC, and the dmPFC. Once the channels were averaged into their respective ROIs, we resampled the timeseries for each area from 3.906 Hz to one sample per second to further smooth our signal and maximize interpretability, with each feature representing 1 s.

#### Outcome measures.

To organize the self-report responses into bins for machine learning analyses, we partitioned the data in a manner that created the most balanced binary distribution of samples. This generated a high group and a low group of nearly equal numbers of participants for each outcome measure. Dichotomization was chosen for several key reasons. First, it aligns with previous neural reference groups studies (e.g., refs. 9–12), ensuring methodological consistency. Second, from a practical perspective, organizations can benefit more from actionable distinctions (high-risk vs. low-risk) rather than continuous scales. Finally, visual inspection revealed bimodal distributions of the outcomes (*SI Appendix*, Fig. S2), supporting a binary categorization that reflects data structure.

Because the data were split into binary high and low groups as close to evenly as possible for separate outcome measures, the midpoint for each measure varied. The point at which the data were most evenly split for I feel burned out and I feel overwhelmed was 3.5, which produced a ratio of 36 to 31 and 35 to 32 for their respective high and low groups. The other measures required that we split the data at integers to maintain relatively evenly balanced classes, so we had to omit the cases that fell on that midpoint. The midpoint for I need a new or different challenge was 3. After omitting the five participants that responded “3,” the sample size for this prediction model was 62 participants (32 high, 30 low). The final outcome measure, I feel underappreciated, was significantly skewed to the right. The most even split for this measure was at 2, which reduced the sample size for this model to 46 participants (27 high, 19 low). Given the relatively large reduction in sample size, imbalanced classes, and the difficulty of interpreting a split at 2 on a scale from 1 to 7, we decided to omit this measure from further analysis.

After dividing the remaining measures into binary classes, we further balanced the distributions to ensure that the model did not simply learn to predict the majority class. To do so, we upsampled the minority class through randomized repetition of cases. The cases that were repeated changed during each iteration of the process.

### Model Training and Prediction with MTPA.

The goal of this study was to build models that can predict people’s feelings about their careers. Following a neural reference groups approach, we trained an algorithm to recognize patterns of neural activity for people who were labeled as high or low on each measure and then classify new people based on the group with which they show greater neural similarity. To make sure that we were simulating out-of-sample prediction, we used leave-one-out cross-validation (LOOCV) to train and test our model. This process involved leaving out one subject at a time, performing feature selection and model training on the remaining N-1 sample, and then testing the predictive performance of the model on the left-out subject. This cycle was repeated for every participant in the dataset to obtain overall prediction accuracy. We selected LOOCV over k-fold approaches because it is the most exhaustive cross-validation procedure that provides the least biased estimate of model performance and retains the most information for model training (48)—an important advantage given our relatively modest sample size for machine learning.

We also applied “MTPA” (30), an approach developed for this experiment to further reduce the temporal dimensionality of the timeseries. MTPA is similar to multivoxel pattern analysis common in fMRI, but rather than selecting for and analyzing a pattern of voxel activations using a spatial searchlight, MTPA applies a “temporal searchlight” to select for timepoints within a timeseries that are most informative for category classification. This temporal searchlight was performed with elastic net feature selection—a regularized regression technique that combines penalties from lasso (L1) and ridge (L2) regression to determine the most effective features. We used MATLAB’s *lassoglm* function with its default parameters, which automatically selects the regularization parameter, lambda, through 10-fold cross-validation. The alpha parameter, which determines the relative contribution of the lasso and ridge penalties, was set at 0.9—closer to lasso in order to encourage as much sparsity as possible while still accounting for multicollinearity among predictors.

After performing MTPA on the training set, we reduced the timepoints of the training and testing timeseries to the features selected by the elastic net. Here, we treat elastic net as a wrapper-based feature selector to obtain a fully interpretable set of timepoints for prediction and follow-up stimulus mapping. Rather than using the penalized coefficients directly for classification where shrinkage artifacts could potentially blur interpretability, we followed workflows that separate feature selection from model estimation (49, 50) and fit a logistic regression model to the full selected features. We then used this model to evaluate predictive performance.

We chose logistic regression because of its simplicity and linear framework, though other classification methods including support vector machines, linear discriminant analysis, or k-nearest neighbors may also be applied. The predictive performance of the model was evaluated on the test subject’s timeseries, which had been completely held out during both the elastic net and logistic regression phases of analysis. This entire process was repeated for each subject under LOOCV, and predictive performance was averaged across all subjects. To improve estimation stability and account for stochastic variation introduced by class balancing during training, the full LOOCV process was then repeated 50 times and averaged. Analyses were performed independently for each measure and ROI.

### Significance Testing.

Permutation testing was conducted to assess the statistical significance of our observed prediction accuracies. We took a maximal statistic approach (51–53) to generating a null distribution and correcting for multiple comparisons, which controls the familywise error rate while accounting for the inherent interdependencies among ROIs and outcome measures. This entailed running the same MTPA-neural reference groups approach described above 1,000 times with shuffled labels across all nine outcome and ROI combinations. For each permutation, the highest accuracy across all nine tests was recorded, yielding a null distribution of maximal values. Final *P*-values were computed as the proportion of permuted maxima that equaled or exceeded the corresponding observed accuracy.

### Subjective Stimulus Coding and Analysis.

Because MTPA selects for certain features that are predictive of between-group differences, we can directly compare these features (i.e., timepoints) to corresponding timepoints in the video. This allows us to explore which aspects of the stimulus are associated with moments of differential neural responses. To test this, we enlisted trained research assistants to code the video. The coders were blind to the study’s hypotheses and research questions to avoid bias, but they were provided with detailed instructions on how to rate different aspects of the video. For each second of the video, the research assistants marked whether the speaker was discussing, embodying, or alluding to the theme on a binary scale. Themes of interest included whether the speaker was referencing work-related reward, anecdotes and stories, negative-valenced topics, positive-valenced topics, social life, personal life and the self, and their emotions.

We then calculated the probability that people were discussing a given topic or theme in the video during the highly selected timepoints versus timepoints that were not selected at all. Top timepoints were defined as the most frequently selected 25 timepoints because this was the number of timepoints that were reliably selected during most tests. Nonselected timepoints were those that were never selected by the MTPA. To account for the hemodynamic lag inherent in fNIRS acquisition, we shifted the feature set backward by 3 s prior to analysis.

To assess the strength of these feature-to-stimulus associations, we performed circle-shift permutation testing. This involved randomly shifting the binary indicator for top-selected timepoints by a random interval between 1 s and the duration of the video minus 1 s and recalculating the odds ratio. This process was repeated 10,000 times creating a null distribution, and statistical significance was assessed by calculating the proportion of null values equal to or greater than the observed odds ratio.

## Supplementary Material

Appendix 01 (PDF)

## Data Availability

Anonymized analysis code, datasets, materials and stimuli data have been deposited in OSF (10.17605/OSF.IO/39P8M) (54).
