# Supplementary material for: Neural predictors of hidden, persistent psychological states at work
Source: Proc Natl Acad Sci U S A. 2025 Oct 13;122(42):e2504382122. doi: 10.1073/pnas.2504382122 (PMC12557504; doi:10.1073/pnas.2504382122)
Supplement: Supplementary file 1 — Appendix 01 (PDF) [file pnas.2504382122.sapp.pdf]

PNAS

Supporting Information for

Neural predictors of hidden, persistent psychological states at work

Bear M. Goldstein[1], Shannon M. Burns[2], Ashley L. Binnquist[1], Macrina C. Dieffenbach[1], Csaba Konkoly[3], Shira Abramowitz[4], and Matthew D. Lieberman[1]

[1]Department of Psychology, University of California, Los Angeles, CA 90095, [2]Department of Psychological Science and Neuroscience, Pomona College, Claremont, CA 91711, [3]Hum Capital, Los Angeles, [4]Summit Impact, Summit Series L.L.C.

Bear M. Goldstein, Matthew D. Lieberman
Email: beargoldstein@ucla.edu, lieber@ucla.edu

This PDF file includes:

Supporting text
Figures S1 to S2
SI references

Other supporting materials for this manuscript include the following:

Movie
Datasets
Software

Psychometric Evaluation of Outcome Measures

We examined four single-item self-report measures to determine whether they captured separable psychological constructs or warranted aggregation into a composite measure. Our a priori intention was to treat these outcomes as distinct aspects of aversive work-related experience, so we conducted inter-item correlation analyses to empirically assess their relationships. The measures were: "I feel burned out," "I need a new or different challenge," "I feel underappreciated," and “I feel overwhelmed.” Although the “I feel underappreciated” measure was excluded from analysis due to its significant positive skew, we include it here for transparency.

We computed Pearson correlations among the four measures. Correlations between items were modest ($r = .14–.42$; mean $r = .32$), suggesting the items were related but not redundant (Fig. S1). Cronbach’s α for all four items was .66 – below the conventional threshold of .70 cited as a minimum for treating items as a scale (1) – indicating insufficient cohesion to justify combining the four measures into a composite.

We also examined the distribution of responses to assess item characteristics and suitability for predictive modeling. All four items exhibited non-normal distributions with varying degrees of skew as well as bimodality, motivating the decision to binarize and balance the samples for subsequent machine-learning analyses (Fig. S2).

Questionnaire

Name:

____

What is the name of your company?

____

What is your current position there?

____

How many years have you worked in your current position?

____

How many years have you worked as an executive overall?

____

We would like to know more about how you feel about your work. Please indicate the extent to which you agree with each of the statements below (as they apply to your feelings at work).

	Strongly disagree (1)	Disagree (2)	Somewhat disagree (3)	Neither agree nor disagree (4)	Somewhat agree (5)	Agree (6)	Strongly agree (7)
I feel engaged	○	○	○	○	○	○	○
I feel that I am thriving	○	○	○	○	○	○	○
I feel burned out	○	○	○	○	○	○	○
I need a new/different challenge	○	○	○	○	○	○	○
I feel underappreciated	○	○	○	○	○	○	○
I feel overwhelmed	○	○	○	○	○	○	○
I feel fulfilled	○	○	○	○	○	○	○

What is your age?

____

What is your self-identified gender?

○ Male

○ Female

○ Other

Your session is recorded using video and photo cameras.

Please indicate which of the following you are comfortable with (select as many as apply):

- ☐ Audio recording only
- ☐ Photographs
- ☐ Video (for data analysis purposes only)
- ☐ Video for presentation purposes--at scientific conferences
- ☐ Video for presentation purposes--in non-scientific talks
- ☐ I am comfortable with you using recordings in all of the ways described here

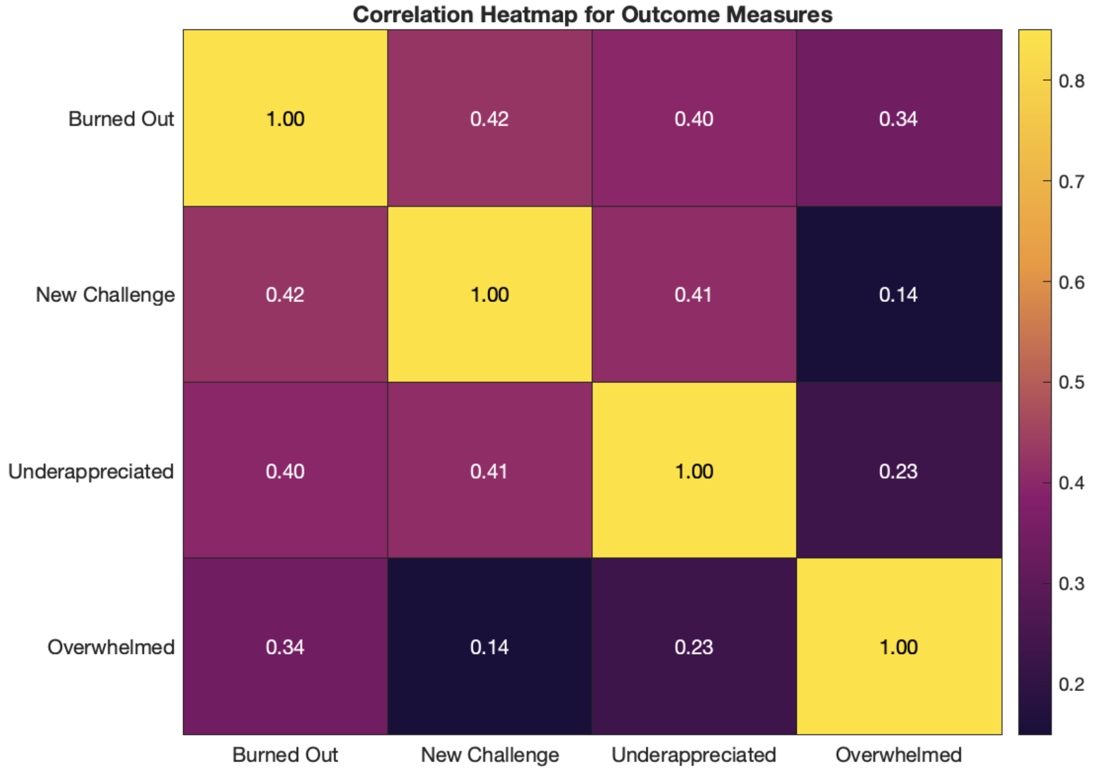

Fig S1. Correlation heatmap for outcome measures.

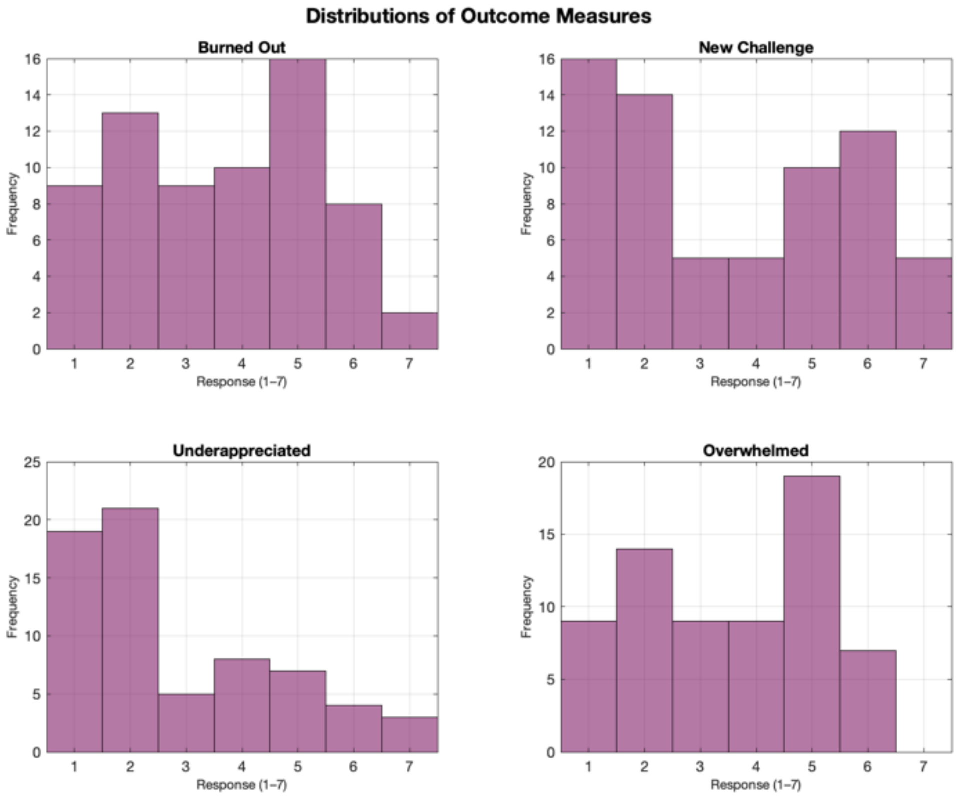

Fig S2. Distributions of individual outcome measures. Responses were on a Likert-type scale from 1 to 7.

SI References

1. J. C. Nunnally, I. H. Bernstein, *Psychometric theory* (McGraw-Hill, 1994).
